# Tweeting birds: online mentions predict future citations in ornithology

**DOI:** 10.1098/rsos.171371

**Published:** 2017-11-01

**Authors:** Tom Finch, Nina O'Hanlon, Steve P. Dudley

**Affiliations:** 1 RSPB Centre for Conservation Science, The Lodge, Sandy SG19 2DL, UK; 2 Conservation Science Group, Department of Zoology, University of Cambridge, Cambridge CB2 3EJ, UK; 3 Environmental Research Institute, University of the Highlands and Islands, Thurso KW14 7EE, UK; 4 British Ornithologists’ Union, PO Box 417, Peterborough PE7 3FX, UK

**Keywords:** altmetrics, citations, social media, Twitter, impact, ornithology

## Abstract

The rapid growth of online tools to communicate scientific research raises the important question of whether online attention is associated with citations in the scholarly literature. The Altmetric Attention Score (AAS) quantifies the attention received by a scientific publication on various online platforms including news, blogs and social media. It has been advanced as a rapid way of gauging the impact of a piece of research, both in terms of potential future scholarly citations and wider online engagement. Here, we explore variation in the AAS of 2677 research articles published in 10 ornithological journals between 2012 and 2016. On average, AAS increased sevenfold in just five years, primarily due to increased activity on Twitter which contributed 75% of the total score. For a subset of 878 articles published in 2014, including an additional 323 ornithology articles from non-specialist journals, an increase in AAS from 1 to 20 resulted in a predicted 112% increase in citation count from 2.6 to 5.5 citations per article. This effect interacted with journal impact factor, with weaker effects of AAS in higher impact factor journals. Our results suggest that altmetrics (or the online activity they measure), as well as complementing traditional measures of scholarly impact in ornithology such as citations, may also anticipate or even drive them.

## 1. Introduction

Whatever the motivation for publishing scientific research, articles should be discovered and read by some target audience. In the Internet age, the mode of publishing, sharing, finding and reading scientific research is evolving [1–3]. Online media channels, including blogging sites and the mass social media networks such as Twitter, have fast become important communication channels for scientists to generate and discuss ideas, find collaborators and disseminate research both within their own communities and to the general public [4]. In addition, various stakeholders (e.g. policy-makers and funders) are now active on—or paying increasing attention to—social media, broadening the potential impact of activity on these platforms.

As individual digital articles supersede paper journal issues, it becomes possible to track not only how often an article is cited in the academic literature, but the real-time attention it receives online. This has led to an increasing interest in alternative metrics, or ‘altmetrics’, which count citations from the social Web [5]. The Altmetric Attention Score (AAS, ©Altmetric) is one method that quantifies the attention received by an article on various online media platforms, including mainstream news, public policy documents, blogs, Wikipedia and social media networks. It has been promoted as a quick and easy way of judging online engagement with research outputs, both individually and in the context of other articles published in the same journal or timeframe (though it cannot distinguish between positive and critical attention [6,7]). The Altmetric ‘dashboard’ also provides a useful tool for tracking (and then contributing to) online conversations surrounding a research output. Although other metrics are available (for example, some publishes make available the number of PDF downloads, or the number of mentions on a particular online platform), the AAS is the altmetric of choice among the major natural science academic publishers.

Weak positive correlations between social media mentions and future citations [5,8–10] suggest that online activity may anticipate or drive the traditional measure of scholarly ‘impact’. Online activity also promotes engagement with academic research, scholarly or otherwise, increasing article views and PDF downloads of *PLoS ONE* articles, for example [11,12]. Thus, altmetrics, and the online activity they represent, have the potential to complement, pre-empt and boost future citation rates, and are increasingly used by institutions and funders to measure the attention garnered by the research they support [13].

The extent to which scientists from different disciplines actively use online platforms for science communication varies [5,14], but its use in the field of ornithology is growing, in part due to the active promotion of the ‘ornithology' hashtag [15]. Ornithology encompasses a diverse community of academics, practitioners, amateur naturalists, birdwatchers and interested members of the public, so the potential for Web-driven ‘non-scholarly’ impact is clear. Here, we explore how AASs for research articles published in ornithological journals have evolved since 2012, describing variation over time and across journals in the ‘raw’ scores as well as the contribution of different online platforms. We then test whether high AASs are associated with more citations in the peer-reviewed literature. We focus explicitly on ornithological research, so our findings should not be extrapolated to broader fields of research. While previous studies have conducted similar analyses for articles published in generalist ecological journals [8], we are unaware of any study of the relationship between online activity and citations for articles in a more specialist field.

## 2. Material and methods

We collated Altmetric data on the raw AAS and number of mentions in each online ‘stream’ from the Altmetric dashboard for 6510 articles published in 10 ornithological journals, including the ‘top five’ (based on 2015 journal impact factor) as well as key ornithological society journals (table 1). The 2015 journal impact factor of these titles ranges from 0.418 to 2.192, reflecting their specialization.

**Table 1.** Summary of the 10 ornithology journals included in analysis, ordered by 2015 journal impact factor (JIF). *n* gives number of articles per journal in the analysis of all data (2012–2016) and the citation analysis (2014). Eight journals had dedicated Twitter accounts by 31 May 2017, with the earliest accounts formed in 2011 and supporting more than 10 000 followers.

journal	2015 JIF	*n* (2012–16)	*n* (2014)	Twitter handle	Twitter since	Twitter followers (31 May 2017)
*Journal of Avian Biology*	2.192	348	71	@avianbiology	2012	6670
*The Auk*	1.871	304	45	@AukJournal	2014	4910
*Ibis*	1.804	378	75	@IBIS_journal	2011	10 800
*Journal of Field Ornithology*	1.514	150	26	@FieldOrnith	2015	2360
*Condor*	1.427	239	24	@CondorJournal	2014	4445
*Journal of Ornithology*	1.419	312	125	—	—	—
*Emu*	1.035	137	34	@EMUJournal	2016	210
*Bird Study*	0.888	315	56	—	—	—
*Wilson Journal of Ornithology*	0.553	394	78	@WilsonOrnithSoc	2013	3660
*Ostrich*	0.418	90	21	@OstrichJAO	2016	255

We then conducted a search of the Scopus database (5 September 2017) using the Elsevier API to collect, for each unique DOI in the Altmetric dataset, publication year, publication type and citation count. We restricted our data to ‘articles’ published between 2012 and 2016, the period over which Altmetric has been available, resulting in 2667 publications with paired Altmetric and citation data. The number of articles included for each of the 10 journals ranged from 90 to 394 (table 1).

### 2.1. Patterns in Altmetric Attention Scores

We first described variation in AAS across years and journals using linear models, with either publication year, journal identity or 2015 journal impact factor as the explanatory variable. Given the strong positive skew in AAS, it was log-transformed for all analyses (values described in the text are original, untransformed values).

### 2.2. Which online platforms drive variation in Altmetric Attention Score?

AASs are measured across various media sources (or ‘streams’); each unique mention found for a research article is collated and contributes to the research article's AAS. Each stream has a ‘value per online mention’, with mentions from different streams weighted based on the impact of their respective audience (table 2). These values are further adjusted by correcting for the ‘reach’, ‘promiscuity’ and ‘bias’ of online attention. For example, on Twitter ‘re-tweets’ have a lower value of 0.85 compared to original posts, while national news outlets have a greater contribution score than smaller, niche publications. We were not able to account for these adjustments, having access only to the number of mentions per stream, so instead simply weighted the absolute number of mentions per stream according to the values in table 2, to determine the approximate contribution of each source to an article's overall AAS.

**Table 2.** The weighting of each scoring stream contributing to a research article's AAS. Note, platforms such as Instagram, Mendeley, CiteULike, Pinterest do not contribute to an article's AAS.

media stream	weight per mention
news media	8
blogs	5
Wikipedia	3
policy documents	3
Twitter	1
F1000/Publons/Pubpeer	1
Open Syllabus	1
Google+	1
Facebook	0.25
YouTube	0.25
Reddit	0.25

We then used generalized linear models to explore variation across journals and years in the weighted number of mentions per stream. Owing to the over-dispersion of the zero-heavy mention data, a negative binomial error structure with log link was used. Models were constructed separately for each of the four main contributing platforms (Twitter, Facebook, news and blogging) with year and journal impact factor as the explanatory variables.

### 2.3. Is there an association between Altmetric Attention Score and citation count?

Finally, for papers published in 2014 (earlier publications had very low AASs, later papers had very low citation counts), we tested the association between AAS and future citations using two modelling approaches. In order to expand our sample from the relatively low-impact (i.e. infrequently cited) articles published in the 10 focal ornithology journals (*n* = 555), we ran a search of the Altmetric database for the keywords ‘bird’ and ‘avian’. As above we used the Elsevier API to collect citation count data. We included only journals with 10 or more articles each, excluding pathological and veterinary journals and *Science*, which had only a narrow distribution of articles with high citation counts and high AAS. In total, data were available for 323 ornithology articles published in 15 non-specialist journals, with 2015 journal impact factors ranging from 2.183 (*PeerJ*) to 9.423 (*PNAS*).

To test the relationship between AAS and citation count, we used a linear mixed model, with citation count as the dependent variable, the additive and interactive effects of AAS and journal impact factor as independent variables, and a random intercept of journal identity. Owing to strong positive skew, all variables were log-transformed, with a small constant (0.1) added to zero values. Second, to test the relationship between AAS and probability of being cited, we converted citation count to a binary score (1 = yes, 0 = no), and used a generalized linear mixed model with binomial error structure and logit link, with the same model terms as above. Prediction uncertainty (95% confidence intervals) was estimated using parametric bootstrapping (1000 simulations), using the *bootMer* function in the *lme4* R package v. 1.1–13 [16]. All analyses were conducted in R v. 3.4.0 [17].

## 3. Results

### 3.1. Patterns in Altmetric Attention Scores

**Figure 1.**
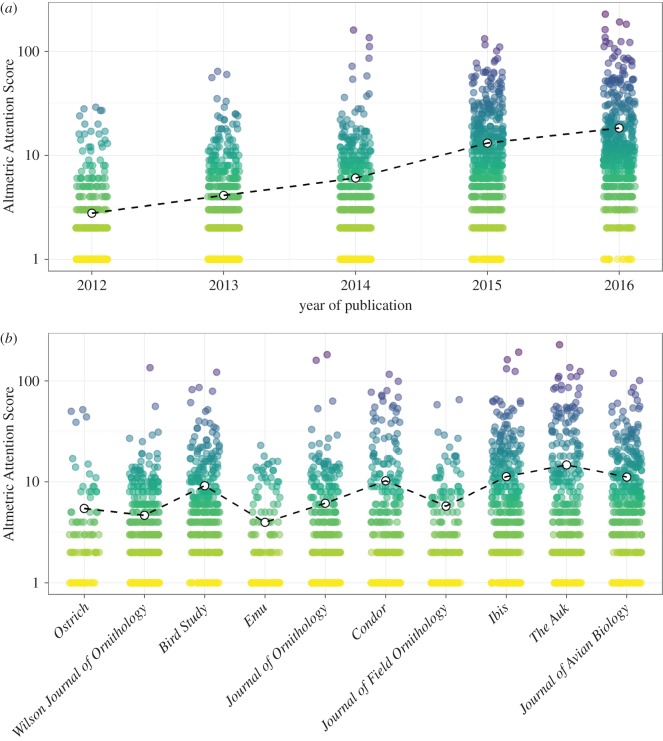
AAS varies according to (*a*) publication year and (*b*) journal identity, with more recent publications in higher impact journals receiving most attention. In both cases, white points connected by dashed lines show mean score. In (*b*), journal titles are arranged in order of increasing impact factor. Note log-transformed *y*-axis.

Raw AAS of articles published in ornithology journals between 2012 and 2016 showed a positive relationship with publication year (linear model, *F* = 1109, d.f. = 1, 2665, *p *< 0.001, *R*^2^ = 0.29), with more recent publications receiving more attention on average (figure 1*a*). In the five years between 2012 and 2016, the mean score (untransformed values) increased nearly sevenfold from 2.8 to 18.3.

Journal identity also explained some variation in AAS, though the explanatory power of this effect was very low (linear model, *F* = 26.81, d.f. = 9, 2657, *p *< 0.001, *R*^2^ = 0.08). The highest mean score was 14.7 (*The Auk*) and the lowest 4.0 (*Emu*). This variation is associated with journal impact factor (which had a positive effect on AAS; linear model, *F* = 111.9, d.f. = 1, 2665, *p *< 0.001, *R*^2^ = 0.04), with articles published in higher impact factor journals (*The Auk*, *Ibis* and *Journal of Avian Biology*) tending to receive more attention on average (figure 1*b*). Nonetheless, there was substantial variation in the AAS of articles published in the same journal.

### 3.2. Which online platforms drive variation in Altmetric Attention Score?

**Figure 2.**
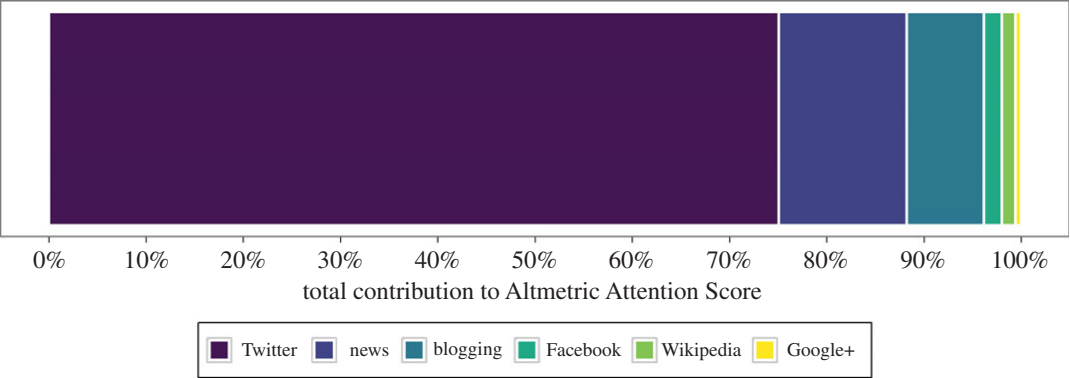
The proportional contribution of each online stream to the total score, calculated as the mean weighted mentions per platform, as a proportion of the sum of weighted mean mentions per platform, averaged across all articles. Other platforms contributed less than 0.01%, and are not shown.

The main online platform contributing to the AAS was Twitter (with the mean weighted number of mentions accounting for 75% of the total), followed by news media (13%), blogging (8%) and Facebook (2%). Other online platforms provided only minor contributions (figure 2).

**Figure 3.**
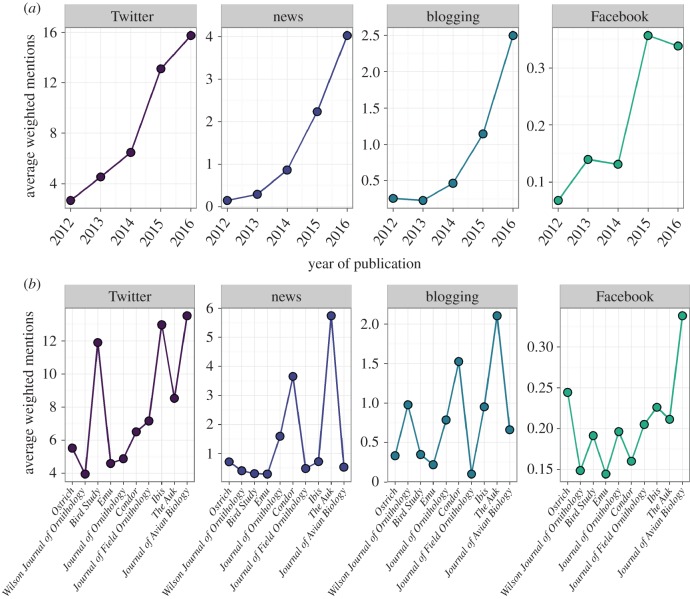
The mean number of weighted mentions per article for each online platform across (*a*) years and (*b*) journals. In (*b*), journal titles are ordered by impact factor (left to right = low to high). Note different *y*-axis scales.

The contribution of these four platforms (Twitter, news, blogging and Facebook) to the overall score varied significantly between years and journals (table 3 and figure 3). Mentions across all platforms have increased year on year, with the rate of increase varying across platforms (figure 3*a*). The number of mentions on Twitter, blogging and Facebook was higher for articles published in higher impact factor journals, and although Twitter remains the most important platform across all journals, news mentions were prominent for articles published in *The Auk* and *Condor* (figure 3*b*).

**Table 3.** Model results for four negative binomial generalized linear models, testing the effect of journal impact factor (JIF) and publication year on the contribution of the four main online platforms to AASs.

platform	variable	estimate	2.5%	97.5%
Twitter	intercept	−884.623	−937.199	−832.174
	year	0.440	0.414	0.466
	JIF	0.360	0.299	0.422
Facebook	intercept	−621.090	−895.124	−629.177
	year	0.308	0.311	0.443
	JIF	0.147	0.162	0.469
news	intercept	−1667.202	−2159.574	−1167.099
	year	0.827	0.579	1.072
	JIF	0.443	−0.153	1.029
blogging	intercept	−1276.000	−1498.542	−1054.870
	year	0.633	0.523	0.744
	JIF	0.347	0.062	0.628

### 3.3. Is there an association between Altmetric Attention Score and citation count?

**Figure 4.**
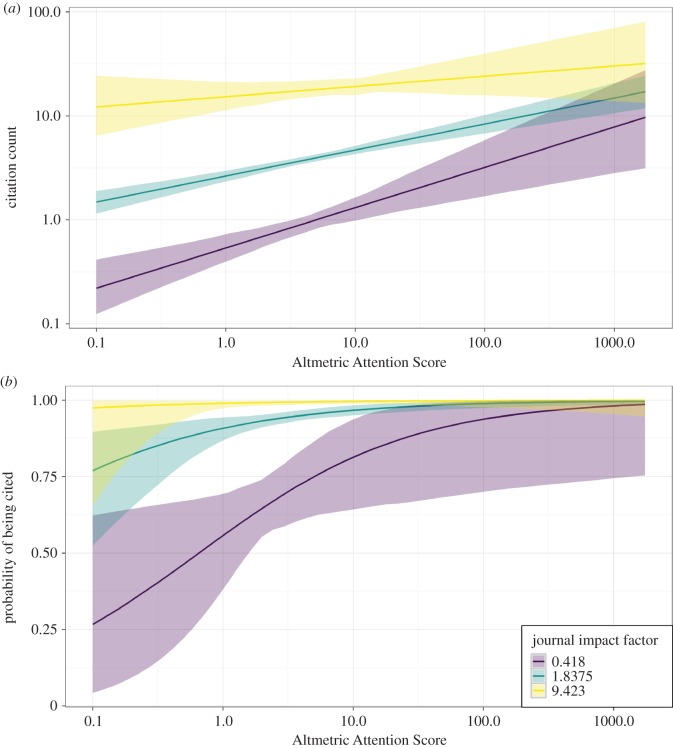
The association between AAS, journal impact factor and (*a*) citation count, and (*b*) probability of being cited. Lines show predicted citation count/probability ± 95% confidence interval for journal impact factors of 0.418 (minimum), 1.838 (median) and 9.423 (maximum). Note log-transformed axes.

Considering only articles published in 2014 (*n* = 878; 555 articles from our 10 focal ornithology journals supplemented with an additional 323 ornithology articles from non-specialist journals), there was a significant effect of both journal impact factor and AAS on the number of citations and the probability of being cited (table 4). Articles receiving higher online attention tended to be cited more, but this effect was weaker for articles in higher impact factor journals which tended to be highly cited regardless of online activity (figure 4). For an article published in a journal with an impact factor of 1.84 (the median among our augmented dataset), the average effect of increasing AAS from 1 to 20 (10th and 90th percentiles, respectively) was 2.9 citations, representing an increase of 112% from 2.6 (2.35–2.92 95% CI) to 5.5 (4.67–6.38) citations per article. The predicted probability of being cited increased by 7% from 0.91 (0.885–0.948) to 0.98 (0.961–0.990). For an article published in a journal with an impact factor of 9.42 (the maximum among sampled titles), this effect represented a smaller and non-significant relative increase of 33% (15.4 (11.74–21.66) to 20.5 (15.77–27.92) citations per article).

**Table 4.** Model results for two mixed models, testing the effect of AAS and journal impact factor (JIF) on the number of citations and the probability of being cited (all continuous variables log-transformed, centred and scaled).

response	variable	estimate	2.5%	97.5%
number of citations	intercept	1.329	1.250	1.408
	AAS	0.306	0.222	0.389
	JIF	0.600	0.521	0.679
	AAS × JIF	−0.072	−0.147	0.003
citation probability	intercept	2.972	2.635	3.428
	AAS	0.571	0.208	0.943
	JIF	0.847	0.550	1.220
	AAS × JIF	−0.036	−0.358	0.275

## 4. Discussion

Our quantitative account of the growing online presence of ornithological research shows that, on average, AASs have increased by 550% in just five years, reflecting growth in the use of online platforms for the promotion of scientific research, including a large uptake within ornithology itself. This has primarily been driven by increased activity on Twitter, the dominant online platform for sharing ornithological research. We also found a correlation between AAS and citation count; moving from the 10th to the 90th percentile of AAS increased citation count by 112% for articles published in journals with the median impact factor.

### 4.1. Altmetrics, citations and impact

Citation based article-level metrics are currently the most widely used to judge an article's scholarly impact. Citation counts are variable and driven by numerous factors (not least the database from which they are taken [18]). Review articles are generally more highly cited than original research articles [19], and several studies of bibliometrics in ecology have illustrated a positive relationship between citation count and the number of words, authors, references and figures [20–22]. These measures may serve as proxies for the quality, novelty or generality of the research or may simply speak to our cognitive biases, with longer author lists or higher-ranking journals signalling ‘prestige’ and generating future citations.

Leaving aside the qualities of the research itself, an article needs to be discovered before it can be cited. Given the increasing importance of online media as a means of locating academic research [3], the positive association between online attention and future citations should not come as a surprise. Our results support the findings of previous studies which have demonstrated links between online attention and citations in broader disciplines [5,8]. Because of the lag between publication and citation, journal-level impact factors are often (mis-)used as a shorthand for article-level impact; however mean citation rates are generally influenced by a small number of highly cited articles, so journal impact factors are often only weakly correlated to article citation rates [23]. Instead, our results suggest that altmetrics might provide an initial and immediate indicator of a research article's future scholarly impact [5], particularly for articles published in more specialist journals.

The correlative nature of this and other studies makes it difficult to establish any causal relationship between online activity and future citations [11]; do often-mentioned articles become cited more because of increased attention, or do more ‘citable’ articles get mentioned more often because of their higher quality? The positive relationship between AAS and citations was strongest for articles in lower impact factor journals. This suggests that articles in higher impact journals, which are usually of interest to a wider general audience, may accumulate citations whether or not they receive online promotion.

Arguably, striving for higher AASs, or at least the wider societal reach they quantify, is a worthy aim in its own right [4,24]. This may be particularly true in the field of ornithology, where the diverse online community includes stakeholders such as conservation practitioners, land managers and birdwatchers, who are likely to be ‘impacted’ by research articles without ever citing them [14]. The impact of a university's research, including its economic and social impact, was an important component in the last research excellence framework in the UK [25], and funding bodies and non-governmental organizations are already exploring how altmetrics can be used to measure outputs and to effectively reach non-academic audiences to incorporate research into policy and practice [26]. These societal impacts are difficult to define and even harder to measure [27], generally depending on some form of field-specific expert assessment of pre-specified objectives. For example, Sutherland *et al*. [28] evaluated the impact of research articles for the conservation of wild bee populations, and found a weak positive correlation between their expert-derived measures of impact and journal impact factors. Bornmann [29] used *PLoS* articles recommended by *F1000 Faculty* members, and found that articles recommended as ‘good for teaching’ were more frequently mentioned in social media networks. Nonetheless, demonstrable links between online activity and societal impact are likely to remain hard to quantify.

### 4.2. Ornithology online

Until recently there was little the individual researcher could do to promote their own research articles. However, with the rise of blogging, social media and other online platforms, scientists can now disseminate their research to a much wider audience. The AAS measures the combined reach of these ‘attention-seeking’ activities.

#### 4.2.1. Twitter

Across disciplines, Twitter is generally considered to be the most important social media network for science communication [8,15,30]. Ornithology has an established Twitter community, driven in part by the early adoption and wide uptake of the dedicated ‘ornithology’ hashtag [15]. Twitter, despite being low-ranking in terms of weight-per-mention, is still by far the largest contributor to AAS in ornithology, and its contribution continues to increase.

Of the 10 ornithology journals considered here, eight currently have dedicated Twitter accounts with two journals, *Ibis* and *Journal of Avian Biology*, being active for the whole period of this study (2012–2016) with over 10 000 and 6000 followers respectively by 2017 (table 1). The social media activity of a journal can contribute significantly to the AAS of its own published articles, and there is a positive correlation between number of followers and the mean weighted number of Twitter mentions among journals (*r* = 0.61, d.f. = 8, *p *= 0.06). Societies such as the British Ornithologists' Union, and research institutes such as the RSPB Centre for Conservation Science can also generate online attention, as can individual researchers [15].

#### 4.2.2. Mainstream news

Mentions in the mainstream news receive the highest weighting for calculating AAS and, after taking this weighting into account, news was the second most important contributor among ornithology articles. The two journals with the highest contribution from news media (*The Auk* and *Condor*) are both based in the USA, and the New World research they generally publish is perhaps more likely to be covered by popular American science news outlets such as Phys.org and ScienceDaily.com.

As with other platforms, news mentions have increased over the last five years, suggesting a growth in the rate at which ornithology articles are covered in these dedicated science news outlets. In 2016, one in every two ornithology articles were mentioned in some online news source on average, compared with just 1 in 50 in 2012.

#### 4.2.3. Blogging

Blogging, the second highest weighted stream and the third most important contributor among ornithology journals, is an area where individual researchers can increasingly influence the online attention received by their articles. There are now a number of established multi-author ornithology blogs (e.g. #theBOUblog, #WaderTales) which allow authors to provide a more accessible summary of their research articles. Blogs also provide an additional online ‘hook’ to promote on social media networks, presenting a more accessible gateway into science and highlighting the wider implications of the research [31]. The rise of blogging is demonstrated by BOU's blog, for which the number of posts has increased from just six in 2012 to 56 in 2016 [15] (S.P.D. 2017, communication), with a resultant 25-fold increase in page views.

#### 4.2.4. Other platforms

Facebook is less dynamic than Twitter (hence the lower AAS weighting, table 2), and is used less by scientists professionally [30]. However, given the popularity of this medium it can still provide an important contribution to the AAS of individual research articles and journals.

Wikipedia currently makes only a small contribution to ornithology AAS, reflecting the fact that only 3.4% of articles in our dataset were cited on the online encyclopaedia. This citation rate increases with article age, with 6.6% of those published in 2012 being cited on Wikipedia, compared with only 2.4% of articles published in 2016. In this respect, Wikipedia mentions resemble traditional scholarly citations, which take a few years to build up. Wikipedia is arguably an under-used resource for online engagement and dissemination in ornithology. The English language version currently consists of 5.41 million articles, receiving more than 7.6 billion page views and 3.4 million article revisions per month (http://stats.wikimedia.org; accessed 15 June 2017). Bond [32] argues that ornithologists should embrace the online encyclopaedia—which is 99.5% accurate for pharmaceutical data, for example [33]—and contribute their own research to relevant sections.

### 4.3. Drawbacks and ‘manipulation’ of altmetrics

It would be remiss not to acknowledge the drawbacks of surrogate metrics such as the AAS, which explicitly does not attempt to measure the ‘quality’ of scientific research. Articles can receive online attention for the ‘wrong’ reasons, for example, if they contain mistakes or flaws and these, or any surrounding controversy, are then discussed in blogs (e.g. retractionwatch.com) or on social media. Odd titles or curious topics can also provide increased attention [34]. Some articles go ‘viral’ simply due to being associated with a spectacular image or video; for example, the highest current AAS in ornithology was driven by a remarkable image of an eagle attacking a deer, covered by 42 news outlets but receiving very little social media attention (18 tweeters and 3 Facebook posts; https://www.altmetric.com/details/1775134).

Online engagement may also be superficial in many cases; for example, many highly mentioned articles are behind paywalls, so are unlikely to be engaged with in much detail beyond the actual tweet/post. More recent articles may also have a higher AAS because of the increasing uptake of social media, and because most mentions occur soon after publication [5] (this study). Perverse incentives can arise when simple metrics (whether number of publications, journal impact factors or altmetrics) are given too much weight by hiring and funding committees. Finally, likes and mentions can be purchased in order to increase a paper's AAS, and publishers can subscribe to news agencies which enable them to increase coverage of their research articles by the mainstream news media.

## 5. Conclusion

With the huge amount of time and effort it takes to undertake research and then publish results arising from it within the peer-reviewed literature, formal publication should not be seen as the end point. Publicizing and promoting the final article are also important, delivering potential benefits to the individual, science community and wider society. Much ornithological research output relates to conservation, a field in which online media, in particular Twitter, has an important role to play in the effective communication of research as part of education and outreach [35,36]. The wider societal impact of communicating science at a time of increasing political mistrust of science should be capitalized upon, though we acknowledge that the impacts of social media are not universally positive. The challenge is to maintain accuracy and nuance whilst making one's research visible in an increasingly information-rich world.

## Data accessibility

Data and R scripts for this study have been deposited in Dryad as http://dx.doi.org/10.5061/dryad.tq0qc [37].
